# Understanding the Early Signs of Chronic Disease by Investigating the Overlap of Mental Health Needs and Adolescent Obesity

**DOI:** 10.3934/publichealth.2015.3.487

**Published:** 2015-08-18

**Authors:** Tyler C. Smith, Besa Smith

**Affiliations:** 1 Department of Community Health, School of Health and Human Services, National University, San Diego, California, USA; 2 The University of California, San Diego, USA

**Keywords:** chronic disease management, mental health, child, adolescent, obesity, risk factors

## Abstract

**Objective:**

Childhood obesity has reached epidemic proportions with two to three-fold increases in prevalence in the past three decades. Sedentary lifestyles and nutrition have been linked to these increases though little is known about mental health illnesses in children and teens which may be precursors to negative modifiable health risk factors. The objective of this study was to investigate for a potentially more clinically practical indicator of depression over a multi-item screen in respect to reporting of overweight and obesity in adolescents. This study further investigated modifiers to this association and stability of association.

**Method:**

This cross-sectional study aggregated 2007/2009 California Health Interview Survey data (n = 6,917 adolescents). Univariate analyses of population characteristics and modifiable behaviors with obesity/overweight and depression are presented. Multivariable weighted logistic regression was used to compare the adjusted odds of overweight and obesity for those children with reported depression.

**Results:**

After controlling for gender, race/ethnicity, age, and modifiable behaviors, there was a statistically significant relationship between reported depression and overweight/obesity (OR = 1.24; 95% CI = 1.04, 1.49). This effect size was consistent in hierarchical models overall and stratified by gender.

**Conclusions:**

Overweight and obesity in adolescents should be understood clinically in the context of depression and other mental health illness. This study highlights a routine primary care or parental screening assessment that could indicate tendencies in adolescent boys and girls which may be precursors to overweight or obesity. Further research should be conducted to identify ways for integrating adolescent mental health screens into primary care.

## 1. Introduction

Childhood obesity is at epidemic levels, estimated to affect one in three children and adolescents in the United States [1]. This increase is especially alarming because the prevalence has nearly tripled in the past 3 decades and acts as a strong risk factor for adult obesity as well as early and late onset chronic diseases [1]–[3]. Research aimed at understanding impact of regular physical activity [4], better nutrition [5], and less sedentary practices for lowering the risk of obesity have been prolific[1]. These have fostered aggressive campaigns against behaviors which many consider to be the cause of childhood obesity and in turn may be resulting in the stabilization of the prevalence of obesity in US children though it is still too early to confirm [6].

Psychological correlates of obesity have been studied though generalizability and consistency of findings remains limited prompting calls for further exploration [7]. In a prospective study of depression and adolescent obesity, depression was shown to be a strong predictor [8]. In another study of major depressive disorder (MDD), there was no overall statistically significant association between MDD and obesity though subgroups of the population were found to have significant associations [9]. Frequently however, less severe mental health disorders go undiagnosed in adolescent populations. Therefore using a diagnosis as a clinical indicator of forthcoming weight challenges may be of limited value in a population or primary care setting, or for parents who may be watching for signs of issues to come. The objective of this study was to investigate the association of reported feelings of being depressed with overweight/obesity in a population of adolescents. Further, potential modifiers of this association including age, gender, and physical activity as well as the stability of the measure of the association between depression and overweight/obesity were investigated and are discussed.

## 2. Materials and methods

### 2.1. Population and data sources

Adolescents included in this research participated in the 2007 and 2009 California Health Interview Survey (CHIS), maintained by the UCLA Center for Health Policy Research under IRB protocol 09–05–103–02 [10]. CHIS is a population-based telephone survey of Californians conducted every two years since 2001. A multi-stage sampling design was used to sample both land line and cellular telephone service covering the geography of the state. Within each household, one adult over 18 years of age was randomly selected to participate and one adolescent (12–17 years old) was selected to participate. The sampling design was focused to maximize the ability to provide estimates for most counties and groups of counties with small populations as well as provide estimates for California's overall population including racial and ethnic groups. Interviews were conducted in five languages: English, Spanish, Chinese (Mandarin and Cantonese dialects), Vietnamese, and Korean; chosen based on analysis of 2000 Census data suggesting the languages that would cover the largest number of Californians. Advance letters and incentives were used to enhance participation with investigation of nonresponse bias not shown to affect the level of data representativeness [11]. The current research was conducted using CHIS public use data files for the adolescent survey and falls under the regulations governing public use data. This study was conducted in compliance with all applicable federal regulations governing the protection of human subjects in research.

### 2.2. Overweight and Obesity

Though imperfect, body mass index (BMI) has become a standard indicator of overweight and obesity and was used to assess excess body fat in this population of adolescents through report of height and weight [12], [13]. This analysis utilized age and gender-specific BMI calculated from self-reported height and weight classified into the following categories based on guidelines from the Centers for Disease Control and Prevention: (1) normal weight or underweight (< 85th percentile) or (2) at risk of overweight (85th–94th percentile) or obese (> 95th percentile) [14].

### 2.3. Simple Depression Assessment

Depression was assessed by the participant's response to the following question “The next questions are about how you have been feeling during the past 30 days”… “How often did you feel so depressed that nothing could cheer you up?” Participants could respond “All of the time”, “Most of the time”, “Some of the time”, “A little of the time”, or “Not at all”. For the purposes of this investigation, responses were dichotomized into “no” or “yes” with no being “Not at all” responses and yes representing a composite of all other participant response options. Additional analyses were also conducted using the full five-level Likert-scale response pattern. The question was taken from the section assessing emotional functioning in the previous 30 days.

### 2.4. Covariates

Seeking the most parsimonious model necessitated including only those variables which may be surrogates of a broad spectrum of variable constructs reported to be associated with overweight/obesity or depression. Demographic characteristics considered for multivariable modeling included: sex, which has been associated with depression and BMI [15],[16] age, (years 12 to 17) [15],[17]; and race/ethnicity (White non-Latino, African American non-Latino, Asian non-Latino, Latino, other), which has been associated with depression and BMI [15]–[17]. Additionally, we included health insurance coverage in the past year (yes, no), whether the individual had seen a doctor in the previous year (yes, no), and poverty level (0 – 99% Federal Poverty Level (FPL), (100 – 199% FPL, 200 – 299% FPL, and 300% FPL and above) [15]–[17]. Modifiable health behaviors considered for multivariable modeling included: intake of 5 or more fruits and vegetables per day in the past week, which has been associated with depression though research is unclear as to the association with BMI [18],[19]; household smoking, which has been associated with BMI and childhood emotional problems [20],[21]; total weekly hours utilizing TV/video games, which has been associated with BMI and depression [22],[23]; and physical activity (in a typical week, how many days physically active for 60 minutes or more), which has been linked to BMI and depression [22],[24],[25].

### 2.5. Statistical Analyses

CHIS uses a two-stage geographically stratified random digit-dial sample design for weighted statistical analyses [26] and provides weights to account for sample selection probabilities, non-response biases, and other adjustments designed for variance estimation. In this cross-sectional study of CHIS data, descriptive and univariate analyses, including weighted chi-square statistics of adolescent depressive, demographic, and behavioral characteristics with overweight/obesity were initially conducted. An exploratory multivariable model was conducted to assess multicollinearity, significant associations, and possible confounding while simultaneously adjusting for all other covariates in the model. A hierarchical weighted multivariable logistic regression was conducted to compare the differences in adjusted odds of overweight/obesity while controlling for possible confounders. The first model included only depression as an independent variable; the second model included depression as well as demographic characteristics; the third model included depression and demographic and behavioral characteristics. These models were also repeated stratifying by gender. Data management and weighted statistical analyses were performed using SAS software (version 9.1.3, SAS Institute, Inc., Cary, NC).

## 3. Results

Adolescents with complete covariate data included a total of 3,582 from the 2007 data appended to the 3,335 adolescents from the 2009 data for a total of 6,917 investigated in this analysis. The sample consisted of 51.2 percent male, near equal percent of each age year (12–17), 53.7 percent White non-Latino, only 22 percent reporting the consumption of five fruits or vegetables each day, 96 percent reporting a smoke free home, 92 percent reporting health insurance coverage, and 85 percent reported seeing a doctor in the past year. Over 50 percent reported 5 or more hours of TV viewing or game use each week, nearly one third reported 2 or less days a week with 60 minutes or more of physical activity, and over one half reported 300% or above on FPL (Table 1).

**Table 1.** Weighted Chi-Square Statistics and Odds Ratios for Demographic and Behavioral Characteristics of CHIS Adolescents Participants By Depression in 2007 and 2009.

Characteristic	CHIS Adolescent Participants(*N* = 6,917)*n* (%)			Participants Reporting Depression(*N* = 1,532; 22.2%)n (%)		*p* value^*^	Odds Ratio (95% CI) ^**^
Gender						<0.0001	
Male	3,540	(51.2)		642	(41.9)		--
Female	3,377	(48.8)		890	(58.1)		1.75 (1.49, 2.05)
Age, years						0.0934	
12	1,026	(14.8)		212	(13.8)		--
13	1,204	(17.4)		238	(15.5)		0.78 (0.59, 1.03)
14	1,191	(17.2)		256	(16.7)		0.77 (0.59, 1.01)
15	1,134	(16.4)		248	(16.2)		0.79 (0.61, 1.03)
16	1,203	(17.4)		279	(18.2)		0.98 (0.76, 1.27)
17	1,159	(16.8)		299	(19.5)		0.98 (0.76, 1.27)
Race/Ethnicity						0.007	
White non-Latino	3,715	(53.7)		723	(47.2)		--
Black non-Latino	258	(3.7)		75	(5.1)		1.46 (1.04, 2.05)
Asian non-Latino	643	(9.3)		138	(9.0)		1.07 (0.81, 1.40)
Latino	2,080	(30.1)		542	(35.4)		1.46 (1.23, 1.74)
Other	221	(3.2)		54	(3.5)		1.32 (0.76, 2.29)
Five Fruits/Vegetables Daily						0.0456	
No	5,384	(77.8)		1,242	(81.1)		--
Yes	1,533	(22.2)		290	(18.9)		0.82 (0.67, 1.00)
Smoking in Home						<0 .0001	
No	6,636	(95.9)		1,440	(94.0)		--
Yes	281	(4.1)		92	(6.0)		2.12 (1.47, 3.06)
Uninsured in Previous Year						0.408	
No	6,356	(91.9)		1,392	(90.9)		--
Yes	561	(8.1)		140	(9.1)		1.14 (0.83, 1.55)
Doctor Visit Previous Year						0.090	
No	1,006	(14.5)		237	(15.5)		--
Yes	5,911	(85.5)		1,295	(84.5)		0.81 (0.64, 1.04)
TV/Game Use Weekly (hours)						0.003	
0–2	1,262	(18.2)		245	(16.0)		--
3–4	1,907	(27.6)		377	(24.6)		0.97 (0.77, 1.22)
5–7	1,829	(26.4)		416	(27.2)		1.18 (0.92, 1.51)
≥8	1,919	(27.7)		494	(32.3)		1.39 (1.09, 1.77)
Physical Activity, days >60 minutes						< 0.0001	
0	745	(10.8)		202	(13.2)		--
1	511	(7.4)		143	(9.3)		0.93 (0.66, 1.32)
2	909	(13.1)		242	(15.8)		0.86 (0.63, 1.18)
3	1,121	(16.2)		250	(16.3)		0.86 (0.60, 1.24)
4	853	(12.3)		170	(11.1)		0.68 (0.46, 1.02)
5	1,163	(16.8)		227	(14.8)		0.62 (0.45, 0.85)
6	525	(7.6)		89	(5.8)		0.36 (0.24, 0.55)
7	1,090	(15.8)		209	(13.6)		0.57 (0.39, 0.84)
Federal Poverty Level						< 0.0001	
0 –99% FPL	1,053	(15.2)		308	(20.1)		--
100 –199% FPL	1,179	(17.0)		304	(19.8)		0.82 (0.63, 1.07)
200 –299% FPL	928	(13.4)		230	(15.0)		0.76 (0.59, 0.97)
300% FPL and above	3,757	(54.3)		690	(45.0)		0.55 (0.44, 0.68)

^*^ *p* values based on Pearson Chi-square test of association using sampling weights for variance estimation.

^**^ Unadjusted odds ratios and 95% confidence intervals using sampling weights for variance estimation.

Demographic characteristics by whether participants reported feeling depressed are described in Table 1. Gender, race/ethnicity, eating fruits or vegetables daily, smoking in the house, TV/game use, and physical activity were associated with feelings of depression. Specifically, a proportionately higher burden of depression was found in individuals who were female, 17 years old, Latino, did not consume 5 fruits/vegetables per day in the past week, lived in a house where smoking is allowed, reported viewing 8 or more hours of TV/game use in an average week, and reported no days of greater than 60 minutes of physical activity.

Demographic characteristics by whether participants report height and weight consistent with a BMI indicative of child or adolescent overweight/obesity are described in Table 2.

**Table 2.** Weighted Chi-Square Statistics for Depression, Demographic and Behavioral Characteristics of Adolescents By Overweight/Obesity in 2007 and 2009.

Characteristic	CHIS Adolescent Participants(*N* = 6,917)*n* (%)			Overweight/Obesity(*N* = 1,728; 25.0%)n (%)		*p* value^*^
Depression						0.002
No	5,385	(77.9)		1,287	(74.5)	
Yes	1,532	(22.1)		441	(25.5)	
Gender						< 0.0001
Male	3,540	(51.2)		1,007	(58.3)	
Female	3,377	(48.8)		721	(41.7)	
Age, years						< 0.0001
12	1,026	(14.8)		297	(17.2)	
13	1,204	(17.4)		327	(18.9)	
14	1,191	(17.2)		269	(15.6)	
15	1,134	(16.4)		292	(16.9)	
16	1,203	(17.4)		276	(16.0)	
17	1,159	(16.8)		267	(15.5)	
Race/Ethnicity						< 0.0001
White non-Latino	3,715	(53.7)		744	(43.1)	
Black non-Latino	258	(3.7)		90	(5.2)	
Asian non-Latino	643	(9.3)		99	(5.7)	
Latino	2,080	(30.1)		738	(42.7)	
Other	221	(3.2)		57	(3.3)	
Five Fruits/Vegetables Daily						0.166
No	5,384	(77.8)		1,364	(78.9)	
Yes	1,533	(22.2)		364	(21.1)	
Smoking in Home						0.157
No	6,636	(95.9)		1,642	(95.0)	
Yes	281	(4.1)		86	(5.0)	
Uninsured in Previous Year						0.076
No	6,356	(91.9)		1,554	(89.9)	
Yes	561	(8.1)		174	(10.1)	
Doctor Visit Previous Year						0.860
No	1,006	(14.5)		261	(15.1)	
Yes	5,911	(85.5)		1,467	(84.9)	
TV/Game Use Weekly (hours)						0.376
0 –2	1,262	(18.2)		298	(17.2)	
3 –4	1,907	(27.6)		431	(24.9)	
5 –7	1,829	(26.4)		489	(28.3)	
≥ 8	1,919	(27.7)		510	(29.5)	
Physical Activity, days >60 minutes						0.007
0	745	(10.8)		201	(11.6)	
1	511	(7.4)		135	(7.8)	
2	909	(13.1)		246	(14.2)	
3	1,121	(16.2)		294	(17.0)	
4	853	(12.3)		208	(12.0)	
5	1,163	(16.8)		287	(16.6)	
6	525	(7.6)		89	(5.2)	
7	1,090	(15.8)		268	(15.5)	
Federal Poverty Level						< 0.0001
0 –99% FPL	1,053	(15.2)		389	(22.5)	
100 –199% FPL	1,179	(17.0)		393	(22.7)	
200 –299% FPL	928	(13.4)		257	(14.9)	
300% FPL and above	3,757	(54.3)		689	(39.9)	

^*^ *p* values based on Pearson Chi-square test of association using sampling weights for variance estimation.

Depression, gender, age, race/ethnicity, and physical activity were associated with overweight/obesity. Specifically, a higher burden of overweight/obesity was found in adolescents with the following characteristics: male, 12 years old, Latino, reporting feelings of depression, and reporting no days with greater than 60 minutes of physical activity.

Multicollinearity among variables was assessed with no variables indicating an issue based on a variance inflation level of ≥ 4. Weighted adjusted multivariable logistic regression analysis results are reported in Table 3. After controlling for gender, race/ethnicity, age, daily fruit and vegetable intake, household smoking status, health insurance, recent doctor visits, screen time based on TV/game use, and physical activity, there was a significant relationship between depression and overweight/obesity (OR = 1.28; 95% CI = 1.07, 1.55). Other variables which were independently associated with overweight/obesity included gender, race/ethnicity, age, and physical activity. Table 4 includes nine weighted and adjusted multivariable logistic regression models. The models present a hierarchical and stratified approach to investigating the association between depression and overweight/obesity. Among the full population, measures of effect for this association ranged from 1.33 for the unadjusted model to 1.28 when adjusting for both demographic and behavioral characteristics. Models stratified by gender showed similar results for men and women. When considering all hierarchical and stratified models the measure of association ranged from a low of 1.25 to a high of 1.43.

**Table 3.** Weighted Logistic Regression for Calculation of Adjusted Odds of Overweight/Obesity in Adolescents (2007, 2009).

Characteristic	CHIS Adolescent Participants(*N* = 6,917)*n* (%)			Weighted Adjusted Odds of Overweight/Obesity*AOR (95% CI*) ^*^	
Depression					
No	5,385	(77.9)		1.00	--
Yes	1,532	(22.1)		**1.24**	**(1.04, 1.49)**
Gender					
Male	3,540	(51.2)		1.00	--
Female	3,377	(48.8)		**0.61**	**(0.51, 0.72)**
Race/ethnicity					
White non-Latino	3,715	(53.7)		1.00	--
Black non-Latino	258	(3.7)		**2.31**	**(1.58, 3.39)**
Asian non-Latino	643	(9.3)		**0.64**	**(0.43, 0.95)**
Latino	2,080	(30.1)		**1.83**	**(1.48, 2.26)**
Other	221	(3.2)		1.18	(0.81, 1.71)
Age, years					
12 to 17				**0.92**	**(0.88,0.97)**
Five Fruits/Vegetables Daily					
No	5,384	(77.8)		1.00	--
Yes	1,533	(22.2)		1.01	(0.82,1.23)
Smoking in Home					
No	6,636	(95.9)		1.00	--
Yes	281	(4.1)		1.24	(0.86, 1.80)
Uninsured in Previous Year					
No	6,356	(91.9)		1.00	--
Yes	561	(8.1)		1.02	(0.75, 1.39)
Doctor Visit Previous Year					
No	1,006	(14.5)		1.00	--
Yes	5,911	(85.5)		1.25	(0.98, 1.58)
TV/Game Use Weekly (hours)					
0-2	1,262	(18.2)		1.00	--
3 –4	1,907	(27.6)		1.02	(0.78, 1.33)
5 –7	1,829	(26.4)		1.19	(0.89, 1.59)
≥ 8	1,919	(27.7)		1.11	(0.83, 1.48)
Physical Activity, days >60 minutes					
0 to 7 days				**0.93**	**(0.90, 0.97)**
Federal Poverty Level					
0 –99% FPL to 300% FPL				**0.84**	**(0.77, 1.33)**

^*^ Model additionally adjusted for year 2007 or 2009.

**Table 4.** Weighted Logistic Regression for Calculation of Adjusted Odds of Overweight/Obesity in Adolescents (2007, 2009).

	Weighted Adjusted Odds of Overweight/Obesity*AOR (95% CI*) ^*^			
	Population n = 6,917	Female n = 3,377	Male n = 3,540OR (95% CI)	
Depression (yes/no)				
Unadjusted	1.33 (1.11, 1.58)	1.38 (1.05, 1.82)	1.43 (1.11, 1.85)	
Adjusted for gender, age, race/ethnicity, uninsured, Federal Poverty Level and doctor visits	1.28 (1.07, 1.53)	1.21 (0.91, 1.62)	1.30 (1.01, 1.69)	
Adjusted for above plus daily fruits/vegetables, smoking in house, TV/computer use, and physical activity	1.24 (1.04, 1.49)	1.21 (0.91, 1.62)	1.27 (0.97, 1.67)	

^*^ Model additionally adjusted for year 2007 or 2009.

## 4. Discussion

The prevalence of obesity in US adults has risen to epic proportions over the past several decades causing great concern and considerable attention to be focused on the concurrent, though lesser, epidemic of childhood and adolescent obesity. Numerous efforts are underway to identify genetic predispositions even while other research suggests the environment to be as likely an influential factor in childhood or adolescent obesity [22],[27],[28]. Investigating emerging mechanisms such as shared biologic pathways of depression and overweight/obesity in adolescents are critical towards reducing the burden of this illness. Though trends of reduction in patient-physician time, higher medical costs, limited resources for population screening, and complexities of diagnosing depression in a primary care setting complicate the landscape, to better address the clinically meaningful approach to identifying these comorbid conditions, this study attempted to highlight a simple screen of potential depression that could be used by clinicians or parents to assess comorbid weight and depressive issues. We present a simplified single item depression screen; findings that indicate a stable relationship between depression and overweight/obesity independent of known demographic and health risk behaviors in both adolescent males and females. The stability underscores the portability of this association and the ease with which it may be utilized.

It is very likely that the causal pathways of overweight/obesity and depression proceed in both directions and that the co-occurrence of these two illnesses is higher than perceived. For that reason, it becomes important to understand mental health morbidity, and specifically depression in absence of elevated BMI. Literature suggests that poor eating behaviors and lack of physical activity are associated with depression and these, in turn, are thought to have the largest impact on elevated BMI [29],[30]. Clinical care among adolescents who are depressed but not overweight or obese should focus on what the patient may be doing to cope with their depressive symptoms and whether these alternative coping mechanisms have a negative impact on their health. Published information regarding other health risk behaviors such as alcohol drinking, tobacco use, and risky sexual behavior show these behaviors are also associated with depression [31],[32]. From a research perspective, if adolescents are feeling depressed and not exhibiting behaviors tending towards a specific coping mechanism then understanding this resiliency will offer insight into biological and social mechanisms which may mitigate depressive symptoms from leading to higher BMI and the beginning of a swirl effect of antagonizing interrelated factors associated with chronic diseases. Identifying depression as early as possible and understanding resilience factors will provide guidance for population health interventions to limit the episodic or chronicity of adolescent to adult depression [15].

Understanding the dynamic nature of adolescent obesity in the context of episodic mental health morbidity has many contributing antagonistic and interrelated factors. Framing these important issues are two recent studies highlighting TV, computer, and video game use and their associations with BMI and depression in adolescents [22],[23]. In the study by Bickham et al, the authors concluded that TV was the key element leading towards higher BMI [22]. In the current study, after adjusting for other covariates including depression, gender, race/ethnicity, age, fruit and vegetable intake, smoking in the home, health insurance coverage, recent doctor visits, and physical activity there was no association between aggregated TV, computer, and video game time and overweight/obesity. We were not able to differentiate by screen media types in this study. Further research into the various screen media uses and how these specific experiences are independently associated with depression and elevated BMI should be conducted to better understand the cause and effect of this pathway.

With an attempt to present a parsimonious model, we utilized a single nutrition variable in order to capture the level of parental nutritional involvement and healthy eating that could be assessed with a large nutritional screen. Those reporting eating five fruits or vegetables daily were less likely to report depressive symptoms which is consistent with research suggesting better physical and mental health associated with higher intake of fruits and vegetables. Less intuitive was the finding that eating five fruits or vegetables daily was not associated with overweight/obesity in both unadjusted and adjusted analyses. Additional studies focused on the contribution of nutrition, including total calories, to both overweight/obesity as well as depression is warranted.

The finding that Black non-Latinos and Latinos carried a heavier proportional burden of depression and overweight/obesity was consistent with the adjusted model which found over a two-fold increase in odds of overweight/obesity after adjustment for the covariates included in the model. This increase has been reported elsewhere along with increased associations for depression [16],[17]. Research continues into disparities that may impact this subpopulation from seeking treatment [33],[34], Should trends persist, however, and despite current steps to lessen the burden of both depression and elevated BMI, these illnesses will increase in prevalence based solely on the expansion of this sub-population in California[35].

Age has been shown in previous research to be associated with both depression and BMI in adolescents [16],[17]. Surprisingly, due to the association of increased BMI and depression, age from 12 to 17 appeared to present an inverted association with BMI and depression. Investigation of possible modification of effect over the years of age was not significant at the alpha = 0.10 level. Still these data are consistent with the literature and appear to suggest a diminishing effect of increase in age and decrease in proportions of overweight/obesity whereas aging increases the proportions of adolescents reporting depression [17].

Limitations to this study are notable and should be considered when understanding the implications of these findings. First, these data were cross-sectional in nature and will not allow temporal sequence to be established which would allow for a better causality assessment. A cohort study allowing for a prospective analysis would be a necessary step forward to establish causality. We, therefore, were not able to observe the temporal sequence of the onset of overweight/obesity or depression in these analyses. Findings yielded associations rather than estimates of risk, though results should be considered important regardless of directional cause. Self-reported data are inherently limited and present possible biases though likely non-differential biasing towards the null. Further, self-report depression and BMI are imperfect surrogates for a physician's diagnosis and could yield non-differential misclassification of either resulting in estimates biased towards the null [13]. A clinician diagnosis of a current mental disorder is certainly optimal over a screen though screening in a primary care setting or discussion by a parent can often be complex [36]. Additionally, diagnosed depression may result in these participants receiving anti-depressive medication which may in itself cause weight gain. Another limitation to these data is that they may not be generalizable to the entire US population and do not include information from institutionalized populations or those without phones. Lastly, the goal of this study was to provide some evidence that a simple question could help providers to better assess potential depression. The question was taken from the section assessing emotional functioning in the previous 30 days and has not been validated or proven to be reliable in this population.

Strengths include the ability to use two years of data (2007 and 2009) from a large cross-section of Californians made possible due to public use data files managed and put forth for research by the UCLA Center for Health Policy Research. The size and scope of the survey, broad inclusion of many racial/ethnic minorities, weighting for both sampling and nonresponse, and the many variables assessed for control of confounding help to make the inferences generalizable on a population base for adolescents. Further, the use of robust statistical procedures with the inclusion of weights for unadjusted and adjusted results allowed for increased inferential potential.

In summary, the coexistence of depression and overweight/obesity in adolescents may be part of comorbid conditions that lead to the early onset of chronic diseases. Integration of mental health services within primary care of adolescents may alleviate some of the stigma or barriers to care that overweight or obese children find in the current care continuum. Further, this integration may help to preemptively screen for weight issues in adolescents presenting for care that do not appear to have weight issues at the time but who may present some level of depression in the past month. The single item question of whether the adolescent may be depressed can be asked in pre-visit health assessments or as a simple probe by the primary care physician or other healthcare professional. This may result in improved identification of future weight issues or the identification of mental health challenges in weight challenged adolescents. In either case, early detection will allow for prevention and treatment interventions with the goal of limiting the long-term burden of both weight and mental health issues. Strategies to lessen the burden of both of these illnesses in adulthood must be integrated to achieve reduction in the independent costs of obesity and mental health.
